# Etiologic and prognostic roles of frailty, multimorbidity and socioeconomic characteristics in the development of SARS-CoV-2 infection and related severe health outcomes: systematic reviews of population-based studies

**DOI:** 10.1093/eurpub/ckac129.062

**Published:** 2022-10-25

**Authors:** T Makovski, J Ghattas, B Devleesschauwer, L Carcaillon-Bentata, PHIRI WP5.2working group

**Affiliations:** 1 Department of Non-communicable Diseases and Trauma, Santé Publique France, Paris, France; 2 Institute of Health and Society, Université Catholique de Louvain, Louvain, Belgium; 3 Department of Epidemiology and Public Health, Sciensano, Brussels, Belgium; 4 Department of Translational Physiology, Infectiology and Public Health, Ghent University, Merelbeke, Belgium

## Abstract

The study had 2 objectives, to: 1) evaluate the etiologic roles of frailty, multimorbidity and socioeconomic status on SARS-CoV-2 infection probability, hospitalization, intensive care unit (ICU) admission, mechanical ventilation and COVID-19 related mortality; 2) investigate the prognostic roles of mentioned risk factors on the likelihood of hospitalization, ICU admission, mechanical ventilation, COVID-19 mortality, functioning, quality of life, disability, mental health and work absence. Three systematic reviews were performed, for each risk factor. The reviews shared first screening steps relying on a common population-based approach. Initial search took place on 7 April 2021 in PubMed, Embase, PsycINFO and WHO Covid-19 database. An update was performed for frailty only, on 1 February 2022, due to the scarce literature retained initially. Prospero registration number: CRD42021249444. Initial search retrieved 10 139 records; 411 studies were read in full text. An update for frailty retrieved 565 records. Finally, the total number of included studies was: for multimorbidity, objective 1 N = 2, objective 2 N = 13; frailty, objective 1 N = 2, objective 2 N = 3; socioeconomic characteristics, objective 1 N = 57, objective 2 N = 30. The risk of severe short-term outcomes such as mortality, ICU admission or hospitalization increased with increasing disease burden and socioeconomic deprivation. Literature on long-term impacts was not identified. The evidence indicates a dose-effect association across all risk factors and outcomes. There is a lack of work conducted on population-based representative samples accounting for frailty and multimorbidity. Measures of multimorbidity and frailty were heterogeneous between studies. Most of the studies observing socioeconomic determinants were performed in the USA and the UK; hence the need for more research in different contexts. Further evidence is required in order to estimate the impact of crisis among general population.

